# *Micromelanconis kaihuiae* gen. et sp. nov., a new diaporthalean fungus from Chinese chestnut branches in southern China

**DOI:** 10.3897/mycokeys.79.65221

**Published:** 2021-04-16

**Authors:** Ning Jiang, Qin Yang, Xin-Lei Fan, Cheng-Ming Tian

**Affiliations:** 1 The Key Laboratory for Silviculture and Conservation of the Ministry of Education, Beijing Forestry University, Beijing 100083, China Beijing Forestry University Beijing China; 2 Forestry Biotechnology Hunan Key Laboratories, Central South University of Forestry and Technology, Changsha 410004, China Central South University of Forestry and Technology Changsha China

**Keywords:** *Castanea mollissima*, Diaporthales, DNA phylogeny, *
Melanconis
*, systematics

## Abstract

*Melanconis*-like fungi are distributed in several families of Diaporthales, mainly Juglanconidaceae, Melanconidaceae, Melanconiellaceae and Pseudomelanconidaceae. A new *Melanconis*-like genus of Pseudomelanconidaceae was discovered on branches of Chinese chestnut (*Castanea mollissima*) in southern China, which was confirmed by both morphology and phylogenetic analysis of combined ITS, LSU, *tef1a* and *rpb2* sequences. The new genus *Micromelanconis* is characterized by two types of conidia from natural substrate and manual media of PDA, respectively. Conidia from Chinese chestnut branches are pale brown, ellipsoid, multiguttulate, aseptate with hyaline sheath. While conidia from PDA plates are pale brown, long dumbbell-shaped, narrow at the middle and wide at both ends, multiguttulate, aseptate, and also with hyaline sheath. All Pseudomelanconidaceae species were only reported on tree branches in China until now. More interesting taxa may be discovered if detailed surveys on tree-inhabiting fungi are carried out in East Asia in the future.

## Introduction

Diaporthales, a species-rich order within Sordariomycetes of Ascomycota, is characterized by perithecia with elongate beaks, often forming within stromatic tissues, deliquescent paraphyses, and asci that have a refractive apical annulus (Barr 1978; Rossman et al. 2007; Senanayake et al. 2017, 2018; Fan et al. 2018a; Jiang et al. 2020a). Species of this order inhabit a variety of substrates, including plants, soil, even living animal tissues (Barr 1978; Castlebury et al. 2002; Sogonov et al. 2008; Yang et al. 2020). Most of them are pathogens associated with plant diseases, and the rest are endophytes in healthy plants or saprobes on dead tissues (Crous et al. 2012a; Chen et al. 2016; Norphanphoun et al. 2018; Jiang et al. 2019d; Xavier et al. 2019; Zhu et al. 2020; Yang et al. 2021). Some diaporthalean fungi cause severe forest diseases, so gained attention in forest pathological studies in recent years. For example, *Cryphonectria parasitica* (Cryphonectriaceae) causes chestnut blight worldwide (Rigling and Prospero 2018; Jiang et al. 2019b); *Cytospora chrysosperma* (Cytosporaceae) causes common polar and willow cankers in China (Fan et al. 2020); *Gnomoniopsis smithogilvyi* (Gnomoniaceae) results in European chestnut fruit rot and branch canker (Shuttleworth et al. 2016; Shuttleworth and Guest 2017; Jiang and Tian 2019; Jiang et al. 2020b).

Diaporthales is well classified into families based on morphological and phylogenetic studies (Voglmayr and Jaklitsch 2014; Norphanphoun et al. 2016; Voglmayr et al. 2017; Fan et al. 2018a; Senanayake et al. 2018; Yang et al. 2018a), and up to 32 families were accepted in the order Diaporthales (Jiang et al. 2021). Specimens can be identified to specific level by morphological characters, such as transversely distoseptate brown conidia of *Coryneum* (Jiang et al. 2018b, 2019c; Senwanna et al. 2018); allantoid ascospores and conidia of *Cytospora* (Fan et al. 2020); two-guttulate fusiform conidia of *Diaporthe*-like taxa (Fan et al. 2018a; Yang et al. 2018a, b); stromatic tissues turning to purple in 3% KOH of Cryphonectriaceae species (Chen et al. 2013, 2018); dark acervular conidiomata with conspicuous central column of *Melanconis*-like taxa (Fan et al. 2016; Jaklitsch and Voglmayr 2020).

*Melanconis*-like taxa are distributed in several families of Diaporthales, mainly Juglanconidaceae, Melanconidaceae, Melanconiellaceae and Pseudomelanconidaceae, which are four morphologically similar clades in distinct phylogenetic clades within this order (Fan et al. 2018b). Species of these four families are usually discovered on branches of Betulaceae, Juglandaceae and Fagaceae, but they are not strong pathogens (Wehmeyer 1937; Du et al. 2017; Voglmayr et al. 2019).

*Castanea*, commonly known as chestnut trees, is a worldwide genus containing several economic species. Chinese chestnut (*C. mollissima*), is widely cultivated in most of the provinces in China. Previous studies have revealed that seven families (Coryneaceae, Cryphonectriaceae, Cytosporaceae, Diaporthaceae, Erythrogloeaceae, Gnomoniaceae and Pseudomelanconidaceae) of Diaporthales have been reported on branches of *Castanea*. *Coryneum castaneicola*, *C. gigasporum* and *C. suttonii* of Coryneaceae were reported on dead and diseased *Castanea mollissima* branches (Jiang et al. 2018b). *Aurantiosacculus castaneae*, *Cryphonectria neoparasitica*, *C. parasitica* and *Endothia chinensis* of Cryphonectriaceae were confirmed to be *Castanea mollissima* canker pathogens (Jiang et al. 2019b). *Cytospora ceratospermopsis*, *C. kuanchengensis*, *C. leucostoma*, *C. myrtagena*, *C. schulzeri* and *C. xinglongensis* of Cytosporaceae were reported to be associated with *Castanea mollissima* branch cankers (Jiang et al. 2020c). *Diaporthe eres* of Diaporthaceae was discovered on dead branches of *Castanea mollissima* in Beijing (Yang 2018). *Dendrostoma aurorae*, *D. castaneae*, *D. castaneicola*, *D. chinense*, *D. parasiticum*, *D. shaanxiense* and *D. shandongense* of Erythrogloeaceae were associated with *Castanea mollissima* stem, branch and twig cankers (Jiang et al. 2019a). *Gnomoniopsis chinensis* of Gnomoniaceae caused severe stem and branch cankers only in Hebei Province (Jiang and Tian 2019; Jiang et al. 2020b). *Neopseudomelanconis castaneae* of Pseudomelanconidaceae was discovered on *Castanea mollissima* branches in Shaanxi Province (Jiang et al. 2018a).

In the present study, investigations were conducted in *Castanea mollissima* plantations in Hunan Province of south China. Two *Melanconis*-like specimens were collected on a cultivated chestnut tree. The aim of the present study was to identify the fresh collections and confirm their phylogenetic positions.

## Materials and methods

### Collection, examination and isolation

The fresh specimens of diseased and dead chestnut branches were collected in a *Castanea mollissima* plantation in Hunan Province of south China. Morphological characteristics of the conidiomata were determined under a Nikon AZ100 dissecting stereomicroscope. More than 20 fruiting bodies were sectioned, and 50 conidia were selected randomly for measurement using a Leica compound microscope (LM, DM 2500). Isolates were obtained by removing a mucoid conidial mass from conidiomata, spreading the suspension onto the surface of 1.8% potato dextrose agar (PDA), and incubated at 25 °C for up to 24 h. Single germinating conidium was removed and plated onto fresh PDA plates. Cultural characteristics of isolates incubated on PDA in the dark at 25 °C were recorded, including the colony color and conidiomata structures. Specimens were deposited in the Museum of the Beijing Forestry University (**BJFC**). Axenic cultures were maintained in the China Forestry Culture Collection Centre (**CFCC**).

### DNA extraction, PCR amplification and phylogenetic analyses

Genomic DNA was extracted from colonies grown on cellophane-covered PDA, using a cetyltrimethylammonium bromide (CTAB) method (Doyle and Doyle 1990). DNA was estimated by electrophoresis in 1% agarose gel and the quality was measured using the NanoDrop 2000 (Thermo Scientific, Waltham, MA, USA). Four partial loci, including the 5.8S nuclear ribosomal DNA gene with the two flanking internally transcribed spacer (ITS) regions, the large subunit of the nrDNA (LSU), and the translation elongation factor 1-alpha (*tef1a*) and DNA-directed RNA polymerase II second largest subunit (rpb2) genes, were amplified by the following primer pairs: ITS1 and ITS4 for ITS (White et al. 1990), LR0R and LR5 for LSU (Vilgalys and Hester 1990), EF1-728F and EF2 for *tef1a* (O’Donnell et al. 1998; Carbone and Kohn 1999), and RPB2-5F and fRPB2-7cR for *rpb2* (Liu et al. 1999). The polymerase chain reaction (PCR) conditions were as follows: an initial denaturation step of 5 min at 94 °C, followed by 35 cycles of 30 s at 94 °C, 50 s at 48 °C (ITS, LSU) or 54 °C (*tef1a*) or 55 °C (*rpb2*), and 1 min at 72 °C, and a final elongation step of 7 min at 72 °C. PCR products were assayed via electrophoresis in 2% agarose gels. DNA sequencing was performed using an ABI PRISM 3730XL DNA Analyser with a BigDye Terminater Kit v.3.1 (Invitrogen, USA) at the Shanghai Invitrogen Biological Technology Company Limited (Beijing, China).

For phylogenetic reconstruction, newly-generated sequences of ITS, LSU, *tef1a* and *rpb2* were initially subjected to BLAST search (BLASTn) in NCBI website (https://www.ncbi.nlm.nih.gov). Then species and their sequences from recently published articles were selected and listed in Table 1 (Crous et al. 2012b; Alvarez et al. 2016; Senanayake et al. 2017; Braun et al. 2018; Fan et al. 2018a; Jiang et al. 2020a; Wang et al. 2020). The sequence alignments of the four individual loci (ITS, LSU, *tef1a* and *rpb2*) were conducted using MAFFT 7 (http://mafft.cbrc.jp/alignment/server/index.html), manually edited in MEGA 7.0.21, and then assembled as a dataset of ITS-LSU-*tef1a*-*rpb2* to infer the phylogenetic placement of our new isolates.

ML and Bayesian analysis were implemented on the CIPRES Science Gateway portal (https://www.phylo.org) using RAxML-HPC BlackBox 8.2.10 (Stamatakis 2014) and MrBayes 3.1.2 (Ronquist and Huelsenbeck 2003), respectively. For ML analyses, a GTR+GAMMA substitution model with 1000 bootstrap iterations was set. MrModeltest 2.3 was used to estimate the best nucleotide substitution model settings for each gene. Bayesian inference (BI) was performed based on the DNA data set from the results of the MrModeltest, using a Markov chain Monte Carlo (MCMC) algorithm in MrBayes 3.1.2. Two MCMC chains were run from random trees for 1000 million generations and stopped when the average standard deviation of split frequencies fell below 0.01. Trees were saved each 1000 generations. The first 25% of trees were discarded as the burn-in phase of each analysis, and the Bayesian posterior probabilities (BPPs) were calculated from the remaining trees. Phylogenetic trees were viewed with FigTree v.1.3.1 and processed by Adobe Illustrator CS5. The nucleotide sequence data of the new taxon have been deposited in GenBank (Table 1).

**Table 1.** Details of the isolates included for molecular study used in this study.

Species	Isolates	GenBank accession numbers			
		ITS	LSU	*tef1a*	*rpb2*
*Apiognomonia errabunda*	AR 2813	DQ313525	NG027592	DQ313565	DQ862014
*Apiosporopsis carpinea*	CBS 771.79	NA	AF277130	NA	NA
*Apoharknessia insueta*	CBS 111377*	JQ706083	AY720814	MN271820	NA
	CBS 114575	MN172402	MN172370	MN271821	NA
*Asterosporium asterospermum*	MFLU 15-3555	NA	MF190062	NA	NA
*Auratiopycnidiella tristaniopsis*	CBS 132180*	JQ685516	JQ685522	MN271825	NA
	CPC 16371	MN172405	MN172374	MN271826	NA
*Aurifilum marmelostoma*	CBS 124928*	FJ890495	MH874934	MN271827	MN271788
*Celoporthe eucalypti*	CBS 127190*	HQ730837	HQ730863	HQ730850	MN271790
*Celoporthe woodiana*	CBS 118785*	DQ267131	MN172375	JQ824071	MN271791
*Chiangraiomyces bauhiniae*	MFLUCC 17-1669	MF190119	MF190064	MF377598	MF377603
*Coniella africana*	CBS 114133*	AY339344	AY339293	KX833600	KX833421
*Coniella eucalyptorum*	CBS 112640*	AY339338	AY339290	KX833637	KX833452
*Coniella fusiformis*	CBS 141596*	KX833576	KX833397	KX833674	KX833481
*Coniella javanica*	CBS 455.68*	KX833583	KX833403	KX833683	KX833489
*Coryneum gigasporum*	CFCC 52319*	MH683565	MH683557	MH685737	MH685729
*Coryneum umbonatum*	D201	MH674329	MH674329	MH674337	MH674333
*Cryphonectria decipens*	CBS 129353	EU442655	MN172386	MN271845	MN271797
*Cryptometrion aestuescens*	CBS 124007*	GQ369457	MN172387	MN271851	MN271798
*Cytospora chrysosperma*	CFCC 89982	KP281261	KP310805	KP310848	KU710952
*Cytospora elaeagni*	CFCC 89633	KF765677	KF765693	KU710919	KU710956
*Dendrostoma aurorae*	CFCC 52753*	MH542498	MH542646	MH545447	MH545405
*Dendrostoma castaneae*	CFCC 52745*	MH542488	MH542644	MH545437	MH545395
*Dendrostoma chinense*	CFCC 52755*	MH542500	MH542648	MH545449	MH545407
*Dendrostoma dispersum*	CFCC 52730*	MH542467	MH542629	MH545416	MH545374
*Dendrostoma mali*	CFCC 52102*	MG682072	MG682012	MG682052	MG682032
*Dendrostoma osmanthi*	CFCC 52106*	MG682073	MG682013	MG682053	MG682033
*Dendrostoma parasiticum*	CFCC 52762*	MH542482	MH542638	MH545431	MH545389
*Dendrostoma qinlingense*	CFCC 52732*	MH542471	MH542633	MH545420	MH545378
*Dendrostoma quercinum*	CFCC 52103*	MG682077	MG682017	MG682057	MG682037
*Dendrostoma quercus*	CFCC 52739*	MH542476	MH542635	MH545425	MH545383
*Dendrostoma shaanxiense*	CFCC 52741*	MH542486	MH542642	MH545435	MH545393
*Dendrostoma shandongense*	CFCC 52759*	MH542504	MH542652	MH545453	MH545411
*Diaporthosporella cercidicola*	CFCC 51994*	KY852492	KY852515	MN271855	NA
*Diaporthostoma machili*	CFCC 52100*	MG682080	MG682020	MG682060	MG682040
	CFCC 52101	MG682081	MG682021	MG682061	MG682041
*Dwiroopa lythri*	CBS 109755*	MN172410	MN172389	MN271859	MN271801
*Dwiroopa punicae*	CBS 143163*	MK510676	MK510686	NA	MK510692
*Foliocryphia eucalypti*	CBS 124779*	GQ303276	GQ303307	MN271861	MN271802
*Foliocryphia eucalyptorum*	CBS 142536*	KY979772	KY979827	MN271862	MN271803
*Gnomonia gnomon*	CBS 199.53	DQ491518	AF408361	EU221885	EU219295
*Harknessia australiensis*	CBS 132119*	JQ706085	JQ706211	MN271863	NA
*Harknessia capensis*	CBS 111829*	AY720719	AY720816	MN271864	NA
*Harknessia ellipsoidea*	CBS 132121*	JQ706087	JQ706213	MN271865	NA
*Harknessia eucalypti*	CBS 342.97	AY720745	AF408363	MN271866	NA
*Holocryphia eucalypti*	CBS 115842*	MN172411	MN172391	MN271882	MN271804
*Immersiporthe knoxdaviesiana*	CBS 132862*	JQ862765	JQ862755	MN271886	MN271805
*Juglanconis juglandina*	CBS 121083	KY427148	KY427148	KY427217	KY427198
*Juglanconis oblonga*	MAFF 410216	KY427153	KY427153	KY427222	KY427203
*Juglanconis pterocaryae*	MAFF 410079	KY427155	KY427155	KY427224	KY427205
*Lamproconium desmazieri*	MFLUCC 15-0870	KX430134	KX430135	MF377591	MF377605
	MFLUCC 15-0872	KX430138	KX430139	MF377593	MF377606
*Macrohilum eucalypti*	CPC 10945	DQ195781	DQ195793	NA	MN271809
	CPC 19421	KR873244	KR873275	NA	MN271810
*Mastigosporella anisophylleae*	CBS 136421*	KF779492	KF777221	MN271892	NA
*Mastigosporella pigmentata*	COAD 2370*	MG587929	MG587928	NA	NA
*Melanconiella ellisii*	BPI 878343	JQ926271	JQ926271	JQ926406	JQ926339
*Melanconiella spodiaea*	MSH	JQ926298	JQ926298	JQ926431	JQ926364
*Melanconis betulae*	CFCC 50471	KT732952	KT732971	KT733001	KT732984
*Melanconis itoana*	CFCC 50474	KT732955	KT732974	KT733004	KT732987
*Melanconis stilbostoma*	CFCC 50475	KT732956	KT732975	KT733005	KT732988
***Micromelanconis kaihuiae***	CFCC 54572*	**MW414473**	**MW414373**	**MW419880**	**MW419878**
	**KH5-4**	**MW414474**	**MW414374**	**MW419881**	**MW419879**
*Nakataea oryzae*	CBS 243.76	KM484861	DQ341498	NA	NA
*Neopseudomelanconis castaneae*	CFCC 52787*	MH469162	MH469164	NA	NA
*Phaeoappendicospora thailandensis*	MFLU 12-2131	MF190157	MF190102	NA	NA
*Prosopidicola albizziae*	CPC 27478	KX228274	KX228325	NA	NA
*Prosopidicola mexicana*	CBS 113529	AY720709	NA	NA	NA
*Pseudomelanconis caryae*	CFCC 52110*	MG682082	MG682022	MG682062	MG682042
*Pseudoplagiostoma corymbiae*	CPC 14161	GU973510	GU973604	GU973540	NA
*Pseudoplagiostoma oldii*	CBS 115722	GU973535	GU973610	GU973565	NA
*Pseudoplagiostoma variabile*	CBS 113067	GU973536	GU973611	GU973566	NA
*Pyricularia grisea*	Ina168	NA	AB026819	NA	NA
*Pyrispora castaneae*	CFCC 54349	MW208108	MW208105	MW227340	MW218535
	CFCC 54351	MW208110	MW208107	MW227342	MW218537
*Sillia karstenii*	MFLU 16-2864	KY523482	KY523500	NA	KY501636
*Sirococcus tsugae*	CBS 119626	EU199203	EU199136	EF512534	EU199159
*Stegonsporium acerophilum*	CBS 117025	EU039982	EU039993	EU040027	KF570173
*Stilbospora longicornuta*	CBS 122529*	KF570164	KF570164	KF570232	KF570194
*Synnemasporella aculeans*	CFCC 52094	MG682086	MG682026	MG682066	MG682046
*Synnemasporella toxicodendri*	CFCC 52097*	MG682089	MG682029	MG682069	MG682049
*Thailandiomyces bisetulosus*	BCC 00018	NA	EF622230	NA	NA
*Tirisporella beccariana*	BCC 38312	NA	JQ655449	NA	NA
*Tubakia seoraksanensis*	CBS 127490*	MG591907	KP260499	MG592094	NA
*Tubakia iowensis*	CBS 129012*	MG591879	MG591971	MG592064	NA
*Ursicollum fallax*	CBS 118663*	DQ368755	EF392860	MN271897	MN271816

Ex-type strains are marked by an asterisk (*) and the strains from this study are in bold.

## Results

The ITS, LSU, *tef1a* and *rpb2*, and combined data matrices contained 624, 867, 513, 865, and 2869 characters with gaps, respectively. The alignment comprised 92 strains, with *Nakataea oryzae* (CBS 243.76) and *Pyricularia grisea* (Ina168) from Magnaporthales as outgroup taxa. The ML analysis yielded a tree with a ln likelihood value of –45806.266577 and the following model parameters: alpha = 0.298226, Π(A) = 0.241173, Π(C) = 0.258552, Π(G) = 0.275145, and Π(T) = 0.225130. For BI analyses, the general time reversible model, additionally assuming a proportion of invariant sites with gamma-distributed substitution rates of the remaining sites (GTR+I+G), was determined to be the best for the ITS, LSU, and *tef1a* loci by MrModeltest, whereas the most appropriate model for the *rpb2* locus was the Tamura-Nei model, additionally assuming a proportion of invariant sites with gamma-distributed substitution rates of the remaining sites (TrN+I+G). The phylogeny resulting from the RAxML maximum likelihood analysis of the combined gene sequence data is shown in Fig. 1. Overall, the topologies obtained from the different phylogenetic analyses were similar, and the best scoring RAxML tree is illustrated here. The bootstrap support values above 50% of maximum likelihood analysis (ML) and Bayesian posterior probability scores (≥0.90) are noted at the nodes.

The *Diaporthales* separates into 32 clades, representing 32 families, and the new isolates were clustered with a well-supported clade (ML/BI = 100/1) in Pseudomelanconidaceae. The two new isolates were different from any known genera in Pseudomelanconidaceae, and represented a new genus (Fig. 1).

**Figure 1.**
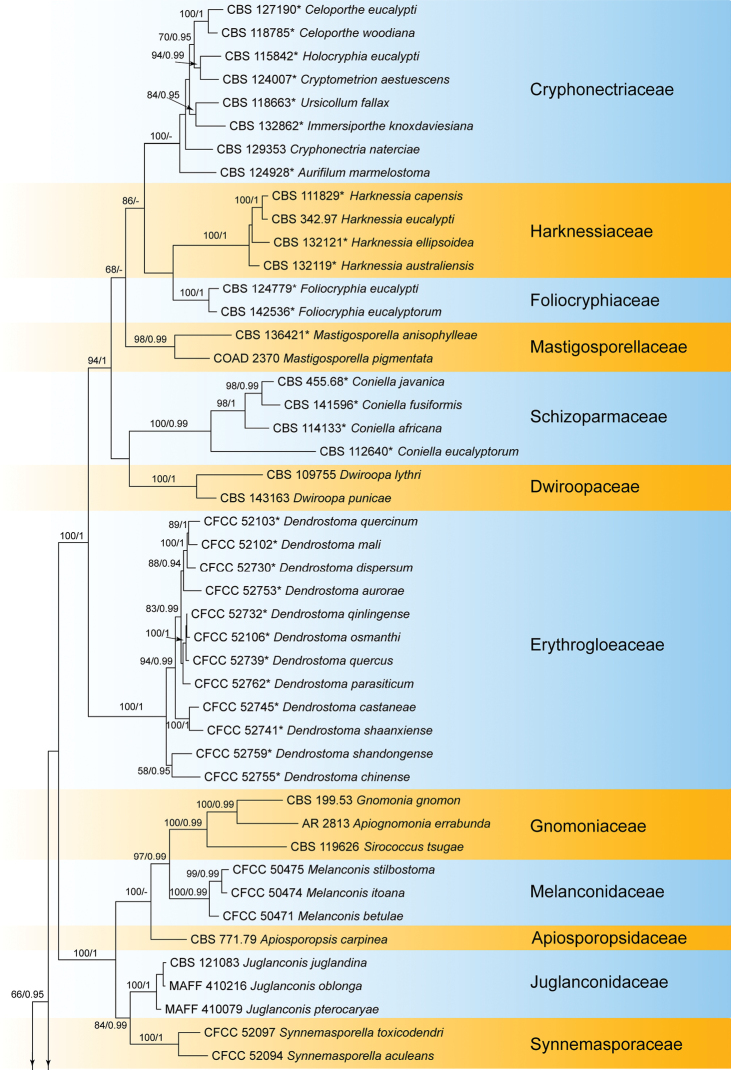
Phylogram of Diaporthales from a maximum likelihood analysis based on combined ITS, LSU, *tef1a* and *rpb2*. Values above the branches indicate maximum likelihood bootstrap (left, ML BP ≥ 50%) and Bayesian probabilities (right, BI PP ≥ 0.90). The tree is rooted with *Nakataea oryzae* (CBS 243.76) and *Pyricularia grisea* (Ina168). New species proposed in the current study is in blue and the ex-type strains are marked with *.

**Figure 1.**
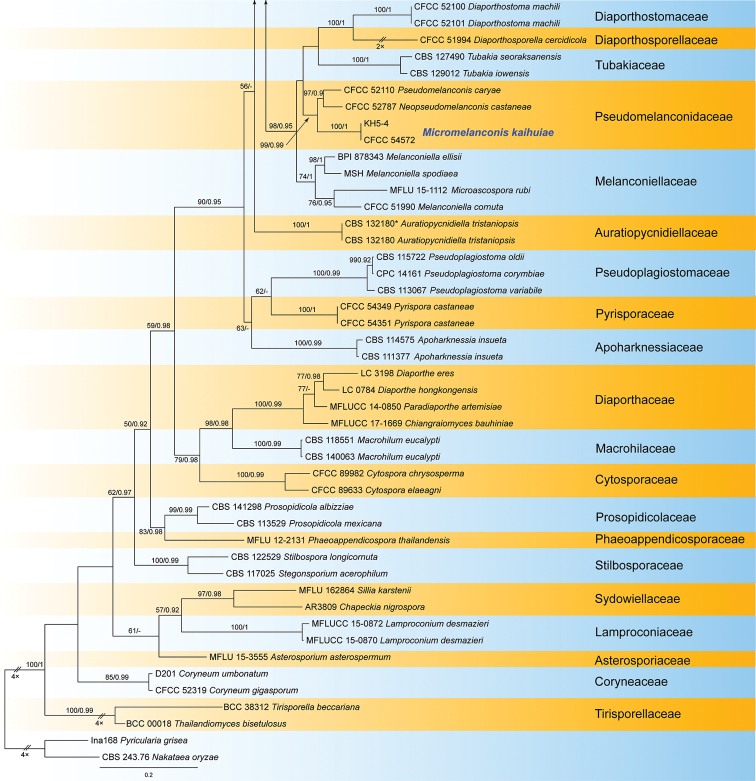
Continued.

### Taxonomy

#### Micromelanconis

Taxon classificationFungiDiaporthalesPseudomelanconidaceae

C.M. Tian & N. Jiang gen. nov.

1FD89DF1-0375-5046-99F4-E2752711B4A5

838927

##### Etymology.

Name derived from micro- and the genus name *Melanconis*.

##### Type species.

*Micromelanconis kaihuiae* C.M. Tian & N. Jiang.

##### Description.

***Sexual morph*:** not observed. ***Asexual morph***: Conidiomata acervular, conspicuous, immersed in host bark to erumpent, covered by brown to blackish exuding conidial masses at maturity. Central column beneath the disc more or less conical. Conidiophores unbranched, aseptate, cylindrical, pale brown, smooth-walled. Conidiogenous cells annellidic, occasionally with distinct annellations and collarettes. Conidia hyaline when immature, becoming pale brown, ellipsoid, multiguttulate, aseptate, with hyaline sheath. Conidiomata formed on PDA after three weeks, randomly distributed, and black. Conidiophores unbranched, septate, cylindrical, pale brown, smooth-walled. Conidiogenous cells annellidic. Conidia pale brown, long dumbbell-shaped, narrow at the middle and wide at both ends, multiguttulate, aseptate, with hyaline sheath.

##### Notes.

*Micromelanconis* is the third genus after *Neopseudomelanconis* and *Pseudomelanconis* in the family Pseudomelanconidaceae (Fig. 1). *Micromelanconis* is united in this family based on the *Melanconis*-like conidiomata, and pale brown conidia with conspicuous hyaline sheath. *Micromelanconis* produces two types of conidia from natural branches and manual media respectively, which differs from *Neopseudomelanconis* and *Pseudomelanconis* (Fan et al. 2018a; Jiang et al. 2018a). Additionally, *Neopseudomelanconis* is characterized by its septate conidia (Jiang et al. 2018a).

#### Micromelanconis kaihuiae

Taxon classificationFungiDiaporthalesPseudomelanconidaceae

C.M. Tian & N. Jiang sp. nov.

608AF9CB-D54A-5D36-9D5F-C38D184B31D6

838928

Figures 2 , 3

##### Etymology.

Named after Kaihui Yang, a Chinese heroine; Kaihui is also the name of the town where holotype was collected.

**Figure 2.**
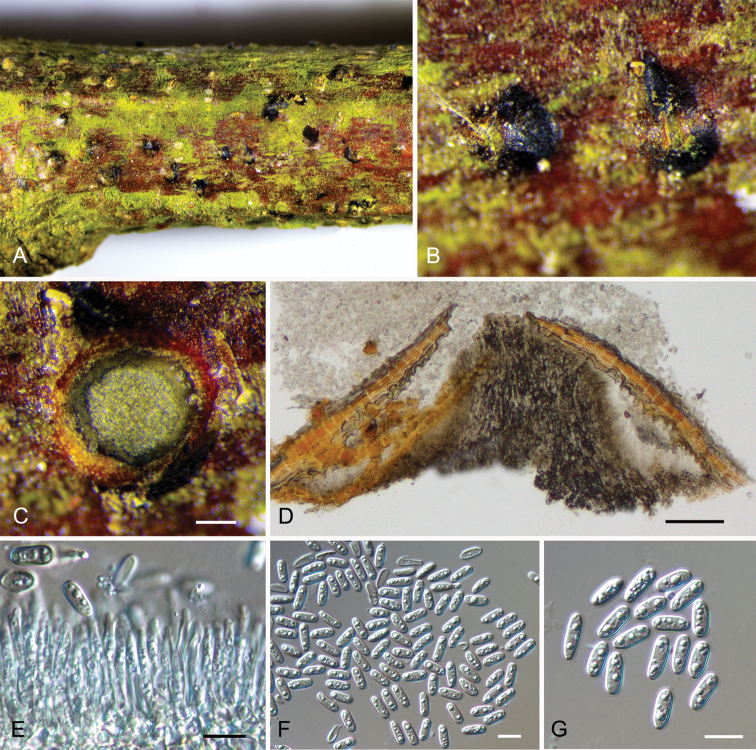
Morphology of *Micromelanconis kaihuiae* on branches of *Castanea mollissima* (BJFC-S1831) **A, B** habit of conidiomata on a branch **C** transverse section of conidiomata **D** longitudinal section through conidiomata **E** conidiogenous cells attached with conidia **F, G** conidia. Scale bars: 100 μm (**C, D**); 10 μm (**E–G**).

##### Description.

***Sexual morph*:** not observed. ***Asexual morph***: Conidiomata acervular, 350–800 μm diam., conspicuous, immersed in host bark to erumpent, covered by brown to blackish exuding conidial masses at maturity. Central column beneath the disc more or less conical. Conidiophores unbranched, aseptate, cylindrical, pale brown, smooth-walled. Conidiogenous cells annellidic, occasionally with distinct annellations and collarettes, 12.4–47.1 × 1.2–3.8 μm. Conidia hyaline when immature, becoming pale brown, ellipsoid, multiguttulate, aseptate, 7.6–10.3 × 3.1–4.1 μm, L/W = 2–3.2, with hyaline sheath, 1 μm.

**Figure 3.**
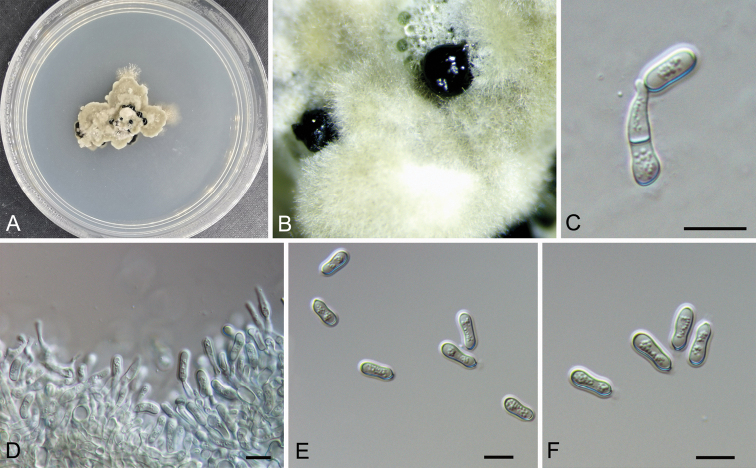
Morphology of *Micromelanconis kaihuiae* on the PDA plate (CFCC 54572) **A** colony on PDA**B** habit of conidiomata formed on PDA**C, D** conidiogenous cells attached with conidia **E, F** conidia. Scale bars: 10 μm (**C–F**).

##### Culture characters.

Colony on PDA at 25 °C irregular, grey olivaceous, margin becoming diffuse, aerial hyphae short, dense, surface becoming imbricate, growth limited and ceasing after two weeks. Conidiomata formed after three weeks, randomly distributed, black. Conidiophores unbranched, septate, cylindrical, pale brown, smooth-walled. Conidiogenous cells annellidic, 9.1–18.5 × 2.5–5.3 μm. Conidia pale brown, long dumbbell-shaped, narrow at the middle and wide at both ends, multiguttulate, aseptate, 10.4–13.5 × 4–5 μm, L/W = 2.3–3.3, with hyaline sheath, 1.5 μm.

##### Specimens examined.

China, Hunan Province, Changsha City, Changsha County, Kaihui Town, chestnut plantation, 40°24'32.16"N, 117°28'56.24"E, 262 m asl, on stems and branches of *Castanea mollissima*, Tian Chengming and Ning Jiang, 10 November 2020 (BJFC-S1831, holotype; ex-type culture, CFCC 54572 = KH5-3). *Ibid.* (BJFC-S1832, KH5-4).

##### Notes.

*Micromelanconis kaihuiae* on *Castanea mollissima* (Fagaceae, Fagales) is phylogenetically close to *Neopseudomelanconis castaneae* on *Castanea mollissima* and *Pseudomelanconis caryae* on *Carya cathayensis* (Juglandaceae, Juglandales) (Fig. 1). All these three species are discovered on tree branches in China, and share similar morphological characters in having pale brown conidia with conspicuous hyaline sheath. *Micromelanconis kaihuiae* and *Neopseudomelanconis castaneae* even share the same host. However, they can be easily distinguished based on conidia shape, color and overall size of conidia (*M. kaihuiae*, pale brown, ellipsoid and aseptate conidia, 7.6–10.3 × 3.1–4.1 μm; pale brown, long dumbbell-shaped and aseptate conidia, 10.4–13.5 × 4–5 μm ***vs.****N. castaneae*, brown, ellipsoid to oblong and septate conidia, 18–21.5 × 4.8–7 μm ***vs.****P. caryae*, pale brown, ellipsoid to oblong and aseptate conidia, 12.5–16 × 4–5 μm) (Fan et al. 2018a; Jiang et al. 2018a). Furthermore, *M. kaihuiae* is separated from *N. castaneae* by 51/490 bp (10.4%) differences in ITS and 12/563 bp (2.1%) differences in LSU, and from *P. caryae* by 56/490 bp (11.4%) differences in ITS and 6/563 bp (1.1%) differences in LSU.

### Key to Pseudomelanconidaceae genera and species

**Table:** 

1	On *Carya* of Juglandaceae, conidia ellipsoid to oblong and aseptate	***Pseudomelanconis caryae***
–	On *Castanea* of Fagaceae	**2**
2	Conidia aseptate	***Micromelanconis kaihuiae***
–	Conidia septate	***Neopseudomelanconis castaneae***

## Discussion

Diaporthales is a well-studied order based on integrated approaches of morphology and phylogeny in recent years (Castlebury et al. 2002; Rossman et al. 2007; Voglmayr and Jaklitsch 2014; Alvarez et al. 2016; Senanayake et al. 2017, 2018; Voglmayr et al. 2017; Braun et al. 2018; Fan et al. 2018a; Jiang et al. 2020a). Thirty-two accepted families are monophyletic and supported by morphological characters; four of them contain *Melanconis*-like fungi, namely Juglanconidaceae, Melanconidaceae, Melanconiellaceae and Pseudomelanconidaceae (Fan et al. 2018a). The *Melanconis*-like fungi were similar in their asexual morph, but well-separated in the phylogeny and their hosts (Voglmayr et al. 2012, 2017, 2019; Fan et al. 2018a, b; Jaklitsch and Voglmayr 2020). In the present study, a new genus and species were clustered in the family Pseudomelanconidaceae (Fig. 1), and differed from the other *Melanconis*-like genera by its long dumbbell-shaped conidia formed on PDA plates.

Hosts are useful taxonomic information in some families of Diaporthales, such as Coryneaceae, Cryphonectriaceae, Erythrogloeaceae and Gnomoniaceae (Voglmayr et al. 2012; Jaklitsch and Voglmayr 2019; Roux et al. 2020; Wang et al. 2020; Yang et al. 2020). Hosts are important to separate *Melanconis*-like genera, *Juglanconis* inhabit *Juglans* and *Pterocarya* of Juglandaceae, *Melanconiella* and *Melanconis* occur only on the plant family Betulaceae (Voglmayr et al. 2012, 2017, 2019; Fan et al. 2018b; Jaklitsch and Voglmayr 2020). *Melanconis* species are discovered only on *Alnus* and *Betula*, while *Melanconiella* occurs in the subfamily Coryloideae with the exception of *M. betulae* and *M. decorahensis* on *Betula* (Voglmayr et al. 2012; Du et al. 2017; Fan et al. 2018a). Species of Pseudomelanconidaceae inhabit *Carya* of Juglandaceae, and *Castanea* of Fagaceae (Fan et al. 2018a; Jiang et al. 2021). More interesting *Melanconis*-like may be revealed by more detailed surveys on tree-inhabiting fungi in the future.

## Supplementary Material

XML Treatment for Micromelanconis

XML Treatment for Micromelanconis kaihuiae
